# Bidirectional associations between maternal controlling feeding and food responsiveness during infancy

**DOI:** 10.3389/fpubh.2022.975067

**Published:** 2022-10-10

**Authors:** Sally G. Eagleton, Lenka H. Shriver, Cheryl Buehler, Laurie Wideman, Esther M. Leerkes

**Affiliations:** ^1^Department of Human Development and Family Studies, UNC Greensboro, Greensboro, NC, United States; ^2^Department of Nutrition, UNC Greensboro, Greensboro, NC, United States; ^3^Department of Kinesiology, UNC Greensboro, Greensboro, NC, United States

**Keywords:** controlling feeding styles, food responsiveness, infancy, bidirectional effects, cross-lagged analysis

## Abstract

Parental controlling feeding styles and practices have been associated with greater food-approaching appetitive behaviors (i.e., food responsiveness) linked to childhood obesity. Recent longitudinal research suggests that this relationship may be reciprocal such that controlling feeding predicts child appetite and vice versa. However, to date no studies have considered these associations during infancy. The current study investigates prospective bidirectional associations between controlling feeding (restriction, pressure, and food to soothe) and infant food responsiveness. Mothers (*N* = 176) reported their controlling feeding and their infant's food responsiveness at infant age 2, 6, and 14 months. A 3-wave cross-lagged panel model was used to test the effect of controlling feeding at an earlier time point on infant food responsiveness at a later time point, and vice versa. Maternal controlling feeding and infant food responsiveness showed moderate stability across infancy. Net of covariates, we observed parent-driven prospective relations between pressuring feeding styles and food to soothe with infant food responsiveness. Pressuring to finish was a significant predictor of increases in food responsiveness from 2 to 6 months (*p* = 0.004) and pressuring with cereal was a significant predictor of increases in food responsiveness from 6 to 14 months (*p* = 0.02). Greater use of situational food to soothe was marginally associated with higher food responsiveness from 2 to 6 months (*p* = 0.07) and 6 to 14 months (*p* = 0.06). Prospective associations between restrictive feeding styles and infant food responsiveness were not observed. Findings point to pressuring feeding styles and food to soothe as potential early life intervention targets to prevent increases in food responsiveness in infancy. Longitudinal research with follow-up in the toddler and preschool years are needed to understand how these associations unfold over time and whether child-driven effects of food responsiveness become apparent as children get older.

## Introduction

Food responsiveness, or a child's tendency to overeat, is an important appetitive behavior indicative of food approach that predicts increased childhood obesity risk. Food responsiveness describes an infant's level of feeding demandingness, responsiveness to milk and feeding cues, and propensity to eat more than provided (1). Individual differences in food responsiveness are observable from early infancy, and elevated food responsiveness has been associated with higher infant weight and rapid infant weight gain (2–4). Food responsiveness is in part genetically determined (5, 6). Although prior studies have shown increases in average food responsiveness scores from 3 months to 15 months (2) and 4 to 11 years (7), significant positive correlations between time points suggest some stability in food responsiveness over time (2, 7). Nevertheless, the expression of this behavioral phenotype depends on interactions with environmental influences (8, 9). Thus, there is great interest in identifying modifiable, early life factors in children's immediate environment that impact food responsiveness.

Parental feeding is believed to be one of the earliest modifiable determinants of children's appetitive behaviors (10) and may contribute to the intergenerational transmission of obesity (11). Feeding practices and styles refer to the attitudes and behaviors surrounding how parents approach the management of what, when, and how much children eat (12, 13). Specifically, controlling feeding such as restricting intake, pressuring to eat, and non-nutritive feeding practices (i.e., feeding to soothe, the use of food as a reward) disregard children's hunger and satiety cues, which over time, can encourage children to eat for reasons besides hunger (e.g., to regulate emotions, in response to visual feeding cues) (12, 14). As such, controlling feeding has been implicated in child obesity risk via its impact on increased food-approaching appetitive behaviors such as food responsiveness.

A substantial body of literature has examined associations between controlling feeding and child weight status. Positive associations between parents' use of food to soothe infant distress, a commonly used controlling feeding practice in infancy, and infant weight have been reported in both cross-sectional and longitudinal studies (15–17). Although studies among infants and young children have generally shown that restrictive feeding is associated with higher child weight status and pressure to eat is associated with lower child weight status, many of these findings come from cross-sectional research and the direction of effects remain unclear (18). For example, do children gain weight because of parents' restriction that may unintentionally prompt children to eat more or do parents restrict intake out of concern that their child is overweight? Given children's appetitive behaviors may mediate the link between parental feeding and child weight (12), controlling feeding is likely influenced by child weight status as well as their appetitive tendencies (19).

Bidirectional associations between parental feeding and child weight have been examined (20–25) and research has increasingly focused on potential bidirectional associations between feeding and appetitive behaviors. A recent systematic review and meta-analysis that included 14 prospective longitudinal studies examining relations between parental controlling feeding and child appetitive behaviors revealed two significant pooled effects (26): higher child food responsiveness predicted increases in restrictive as well as instrumental feeding (i.e., the use of food as a reward). In line with these results, a recent study showed that higher child food responsiveness at 4–5 years predicted an authoritarian feeding style marked by high levels of parental control when children were aged 7–9 years (27). Although pooled analyses were not conducted for longitudinal associations between pressure to eat and food responsiveness or between emotional feeding and food responsiveness in the aforementioned systematic review due to a limited number of studies, 3 of the 14 prospective studies reported significant longitudinal effects. One study found a bidirectional relationship between pressure to eat and food responsiveness such that higher pressure to eat at age 4 predicted lower food responsiveness at age 7 and vice versa (28). In contrast, a study conducted with children 1.5–2.5 years old showed that encouragement to eat (e.g., prompting, praise for eating) was positively associated with children's tendency to overeat 1 year later (29). Two studies reported parent-driven effects of emotional feeding (a measure that encompasses feeding to soothe) on increased child food responsiveness across two time points over a one-year period among children aged 1.5–2.5 years at baseline (29) and across four time points spanning a 3-year period (aged 2–4 years at baseline, 1, 2, and 3 years later) (30).

Taken together, these findings point to the complex and likely bidirectional nature of parent-child feeding interactions, yet longitudinal studies are currently needed, particularly those that formally test bidirectional effects controlling for prior levels of feeding and food responsiveness. Previous longitudinal research examining controlling feeding and food responsiveness has primarily been conducted among children 2 years of age and older. To our knowledge, no studies to date have been conducted among children across the 1st year of life. Given rapid infant weight gain, especially during early infancy (0–6 months), is associated with later obesity and related comorbidities (31, 32), it essential to understand how obesogenic parent feeding and child appetitive behaviors influence one another during this sensitive period of development.

The purpose of the current study is to prospectively examine bidirectional associations between parental controlling feeding (restriction, pressure, and food to soothe) and infant food responsiveness in a community sample of mother-infant dyads using a 3-wave cross-lagged panel model. Based on previous research, we hypothesized that: (1) greater maternal endorsement of a pressuring feeding style and greater use of food to soothe predict increases in infant food responsiveness, and (2) infant food responsiveness predicts increases in maternal restrictive feeding over time.

## Methods

### Participants

Pregnant women were recruited in Guilford County, North Carolina to participate in the Infant Growth and Development Study (iGrow), an ongoing longitudinal study examining prenatal and early life predictors of childhood obesity risk. Recruitment methods included childbirth education classes, Special Supplemental Nutrition Program for Women, Infants and Children (WIC) breastfeeding classes, flyers advertised in OB/GYN clinics, and social media. Eligibility criteria consisted of (1) maternal age ≥18 years, (2) expecting a singleton, (3) written English comprehension, and (4) plans to remain in the region for at least 3 years. Participants in the current study included mother-infant dyads from iGrow cohort 1 (*N* = 176).

### Procedures

During the 3rd trimester of pregnancy, mothers provided written consent and completed online questionnaires using Qualtrics, a popular survey platform. Approximately 1 week after infants' due dates, we obtained infant birth details via phone interviews and confirmed mother's eligibility. Mothers completed online questionnaires again when infants were ~2, 6, and 14 months old. Women were compensated $50 for the prenatal visit, $70 for the 2-month visit, $80 for the 6-month visit, and $90 for the 14-month visit. Data collection for cohort 1 took place between February 2019 and October 2020. This study was approved by the university's Institutional Review Board (protocol #18-0198).

### Measures

#### Maternal controlling feeding

The Infant Feeding Styles Questionnaire (IFSQ) was used to measure mother's controlling feeding styles (33). The IFSQ was originally validated in a low-income sample of Black mothers (33) but has been successfully used with mothers representing a diverse range of sociodemographic characteristics (34–36). To reduce the length of the entire questionnaire battery and thus participant burden, several items were omitted across multiple individual measures. For the IFSQ specifically, mothers completed a subset of 79 of the 83 IFSQ items, which yielded 13 subscales. Mothers rated their behaviors and beliefs around feeding an infant on a 5-point scale. Response options for behavior items ranged from never to always and response options for belief items ranged from disagree to agree. For the current study, we focused on four subscales that are considered controlling: pressuring-finish, pressuring-cereal, restrictive-amount, and restrictive-diet quality. The IFSQ has 20 behavioral items related to feeding solid foods for infants ≥6 months. Consequently, the pressuring-finish (“insist re-try new food refused at same meal”; “praise after each bite to encourage finishing”) and restrictive-diet quality (“I let child eat fast food”; I let child eat junk food”) subscales have two more items at 6 months and 14 months than at 2 months. One of the removed items was from the pressuring-finish subscale (“I try to get my baby to finish his/her breastmilk or formula) and another was from the pressuring-cereal subscale (“I give/gave my baby cereal in a bottle”). Items were averaged to create a summary score for each subscale at each time point with higher scores indicating greater endorsement of the given feeding behavior/belief. The subscales used in the current study had adequate internal consistency reliability: pressuring-finish (five items at 2 months, α = 0.69; seven items at 6 months and 14 months α = 0.70–0.76), pressuring-cereal (four items at all time points, α = 0.78–0.80), restriction-amount (5 items at all time points, α = 0.71–0.78), and restriction diet quality (five items at 2 months, α = 0.74; seven items at 6 months and 14 months, α = 0.74–0.84).

We used the Food to Soothe Scale (15) to measure the controlling feeding practice feeding to soothe. Mothers completed the 6-item situational subscale (e.g., use food to soothe baby in the grocery store, while in the car) and the 3-item state-based subscale (e.g., when you are stressed, tired, nothing else works). Mothers rated their likelihood of using food to soothe for each item on a 5-point scale (never to always) The 6 situational items were averaged to create a summary score at each time point (α = 0.76–0.81) as were the 3 state-based items (α = 0.76–0.80).

#### Infant food responsiveness

The 6-item food responsiveness subscale from the Baby Eating Behavior Questionnaire (BEBQ) (1) was used to assess infant food responsiveness at each postnatal wave. The BEBQ, although originally validated as a retrospective measure of infant appetite focused on the period of exclusive milk feeding (breast or bottle) (1), is commonly used to evaluate infant appetite across the 1st year (4, 37, 38). Mothers rated the extent to which their infant exhibited behaviors that reflect increased feeding demandingness and hunger (“My baby frequently wants more milk than I have given him/her”; “If given the chance, my baby would always be feeding”) on a 5-point scale (never to always), with higher scores indicating higher infant food responsiveness. A mean score was calculated at each time point (α = 0.80 at all waves).

#### Demographic characteristics and covariates

Mothers reported their age, race/ethnicity, educational attainment, individuals residing in the home, income, pre-pregnancy weight and due date prenatally. We calculated an income-to-needs ratio by dividing total annual household income by its corresponding poverty threshold determined by the year in which income is earned and the total number of household members. We used the Poverty Thresholds for 2018 and 2019 published in U. S. Census Reports (39). At a prenatal laboratory visit, trained research staff measured mother's height in duplicate and pre-pregnancy Body Mass Index (BMI; kilograms/m^2^) was calculated using measured height and self-reported pre-pregnancy weight. Approximately 1 week after infant's due date, mothers reported infant sex, birth weight, and infant birth date, which was used to calculate gestational age. At each wave, mothers provided detailed feeding information using a modified version the Infant Feeding Practices Questionnaire Study II (40). Mothers reported the number of feeds that were breastmilk or formula over the past 7 days. At 2 and 6 months, the percentage of feeds as breastmilk was used to categorize infants as exclusively breastmilk fed, exclusive formula fed, or mixed fed (combination of breastmilk and formula). In addition, because all infants had been introduced to solid foods (e.g., complementary feeding) by 14 months, infants were categorized as breastfed (1 = any breastmilk) or not breastfed (0 = no breastmilk) at all three time points.

### Statistical analysis

**Figure 1:**
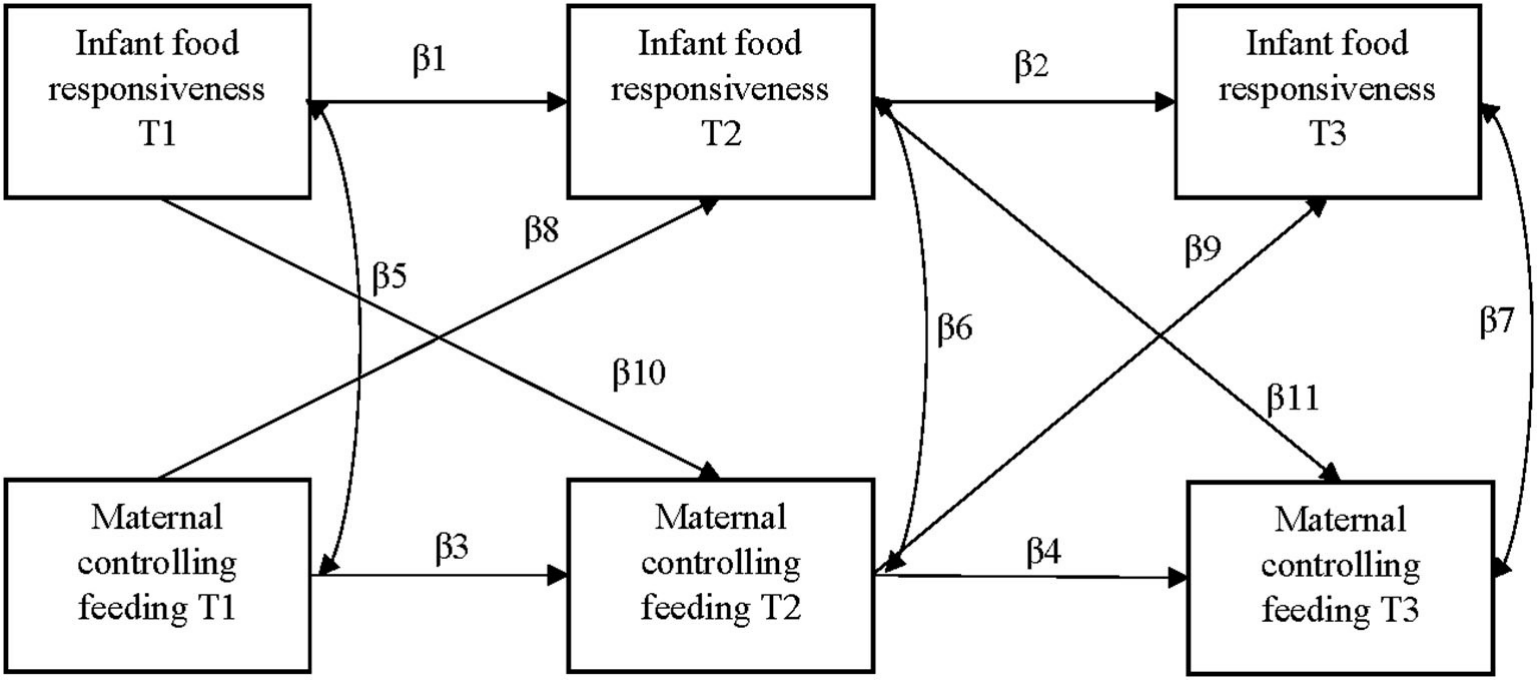
Conceptual model for longitudinal associations between maternal controlling feeding and infant food responsiveness. T1 = infant age 2 months, T2 = infant age 6 months, T3 = infant age 14 months.

Preliminary analyses were conducted using SPSS Version 27 (IBM, Chicago, IL) and univariate statistics were used to describe the sample. Cross-lagged path models were conducted using AMOS Version 27 (IBM, Chicago, IL) to examine associations between maternal controlling feeding and infant food responsiveness at infant age 2, 6, and 14 months. Separate models were conducted for each controlling feeding subscale (i.e., pressure-finish, pressure-cereal, restrictive-amount, restrictive-diet quality, situational food to soothe, and state-based food to soothe). Time invariant covariates collected prenatally (i.e., maternal age, race/ethnicity, measures of socioeconomic status, pre-pregnancy BMI) and infant weight-for-age *z*-score at birth were adjusted for at the first time point. We also examined time invariant covariates in relation to outcome variables at later time points. However, given the small sample size, and for parsimony, if a time invariant covariate was not significantly associated with the outcome at that time point, the path was removed from the model. Covariates that were time varying, which included exact infant age and breastfeeding status at each time point, were adjusted for at their respective time point (e.g., infant age at 2 months was specified on infant food responsiveness and controlling feeding at 2 months). Cross-lagged path models were used to examine the bidirectional associations between controlling feeding and infant food responsiveness across infancy (Figure 1). The use of a cross-lagged analytic model was an ideal design given this type of analysis allows for the simultaneous evaluation of three types of associations: stability coefficients between repeated measures over time (e.g., pressuring feeding at 2 months with pressuring feeding at 6 months), concurrent correlations between controlling feeding and food responsiveness at each time point, and cross-lagged associations that estimate the prospective effect of controlling feeding at an earlier time point with food responsiveness at a later time point, and vice versa. Full information maximum likelihood was used to handle missing data (41). Because small prospective effects between controlling feeding and child appetitive behaviors have been previously reported (26), we interpreted our findings considering both statistically significant (*p* < 0.05) and marginally (*p* < 0.10) significant results.

## Results

### Sample characteristics

Table 1 displays the characteristics of the sample. The mean (*SD*) age of mothers was 29.10 (5.92) years, 54% of mothers identified as non-White, 22% of mothers had a high school education or less, and 56% had overweight or obesity pre-pregnancy. Approximately half of infants were female. Most infants were full-term and had a normal birth weight according to their gestational age. At 2 months, 6 months, and 14 months, 73.5%, 55.9%, and 31.8% of infants were breastfeeding (i.e., fed any breastmilk).

**Table 1:** Sample characteristics and descriptive statistics for primary variables (at baseline unless otherwise noted), *N* = 176.

**Characteristic**	***N***	**% or Mean (SD)**
Maternal age, years	174	29.10 (5.92)
Income to needs ratio	167	3.10 (2.98)
Pre-pregnancy BMI, kg/m^2^	163	28.41 (7.50)
**Pre-pregnancy weight status**		
Underweight	5	3.1%
Normal weight	66	40.5%
Overweight	37	22.7%
Obese	55	33.7%
**Maternal race/ethnicity**		
Non-Hispanic White	81	46.0%
Non-Hispanic Black	57	32.4%
Hispanic/Other/Multiracial	38	21.6%
**Maternal education**		
≤High school diploma/GED	37	21.5%
Some college	40	23.3%
2-year college degree	17	9.9%
4-year college degree	35	20.3%
Post graduate work/degree	43	25.0%
**Infant sex**		
Male	84	50.3%
Female	85	49.7%
Infant gestational age, weeks	169	39.24 (1.44)
Infant weight-for-age z-score at birth	169	−0.03 (1.15)
Exclusive breastmilk, 2 months	151	68 (45.0%)
Exclusive breastmilk, 6 months	145	50 (34.5%)
	**2 months (T1)**	**6 months (T2)**	**14 months (T3)**
Infant age, months	2.24 (0.56)	7.12 (1.39)	15.02 (1.00)
Any breastfeeding	111 (73.5%)	81 (55.9%)	41 (31.8%)
**Maternal controlling feeding**			
Pressuring: finish	2.12 (0.77)	2.28 (0.67)	2.32 (0.74)
Pressuring: cereal	1.77 (0.84)	1.98 (0.99)	1.83 (0.92)
Restrictive: amount	2.91 (1.00)	2.87 (1.04)	2.77 (1.10)
Restrictive: diet quality	3.39 (0.84)	3.84 (0.65)	3.62 (0.84)
Food to soothe: situational	2.54 (0.92)	2.54 (0.97)	2.49 (0.92)
Food to soothe: state-based	2.67 (1.12)	2.77 (1.14)	2.56 (1.10)
Infant food responsiveness	2.72 (0.78)	2.34 (0.72)	2.73 (0.67)

### Concurrent and prospective associations between maternal controlling feeding and infant food responsiveness

Table 2 shows all path coefficients from the cross-lagged analysis depicted in Figure 1. Maternal controlling feeding styles and food to soothe were relatively stable across infancy. In general, there was greater stability in maternal feeding from 6 months to 14 months (β = 0.52–0.67) than from 2 months to 6 months (β = 0.44–0.60). Infant food responsiveness also demonstrated significant stability across infancy (2 to 6 months: β = 0.33; 6 to 14 months: β = 0.27).

**Table 2:** Standardized path coefficients for model shown in Figure 1 (*N* = 176).

	**Path**										
	**Stability coefficients for infant FR**		**Stability coefficients for maternal feeding**		**Concurrent associations of infant FR and maternal feeding**			**Cross–lagged associations Maternal feeding predicting future infant FR**		**Cross–lagged associations Infant FR predicting future maternal feeding**	
Maternal feeding variable	FR T1 → FR T2	FR T2 → FR T3	Maternal feeding T1 → Maternal feeding T2	Maternal feeding T2 → Maternal feeding T3	FR T1 → Maternal feeding T1	FR T2 → Maternal feeding T2	FR T3 → Maternal feeding T3	Maternal feeding T1 → FR T2	Maternal feeding T2 → FR T3	FR T1 → Maternal feeding T2	FR T2 → Maternal feeding T3
Path label in Figure 1	β1 (95% CI)	β2 (95% CI)	β3 (95% CI)	β4 (95% CI)	β5 (95% CI)	β6 (95% CI)	β7 (95% CI)	β8 (95% CI)	β9 (95% CI)	β10 (95% CI)	β11 (95% CI)
Pressuring– finish	0.30*** (0.13–0.42)	0.27** (0.09–0.40)	0.53*** (0.37–0.61)	0.65*** (0.56–0.86)	0.21* (0.03–0.21)	0.08 (−0.03–0.08)	0.16+ (−0.01–0.11)	0.23** (0.07–0.38)	0.16+ (−0.01–0.33)	0.08 (−1.07–1.20)	0.04 (−0.10–0.17)
Pressuring– cereal	0.32*** (0.15–0.44)	0.28** (0.11–0.42)	0.44*** (0.30–0.55)	0.52*** (0.40–0.69)	0.14 (−0.02–0.22)	0.17* (0.00–0.16)	0.10 (−0.04–0.12)	0.14+ (−0.01–0.23)	0.24** (0.05–0.30)	0.00 (−0.15–0.15)	−0.02 (−0.20–0.16)
Restrictive– amount	0.32*** (0.15–0.44)	0.26** (0.08–0.39)	0.60*** (0.47–0.73)	0.67*** (0.63–0.90)	0.14+ (−0.01–0.23)	0.19* (0.01–0.17)	0.06 (−0.05–0.11)	0.12 (−0.03–0.20)	0.09 (−0.05–0.18)	−0.02 (−0.18–0.13)	−0.02 (−0.22–0.16)
Restrictive–diet quality	0.34*** (0.17–0.46)	0.28*** (0.10–0.40)	0.54*** (0.32–0.54)	0.60*** (0.57–0.91)	0.01 (−0.10–0.11)	−0.00 (−0.06–0.06)	−0.16+ (−0.13–0.01)	0.02 (−0.12–0.15)	−0.11 (−0.27–0.05)	0.03 (−0.09–0.14)	0.02 (−0.14–0.17)
Food to soothe– situational	0.32*** (0.15–0.44)	0.25** (0.07–0.38)	0.47*** (0.36–0.64)	0.64*** (0.49–0.74)	0.16+ (−0.00–0.23)	0.04 (−0.07–0.10)	0.03 (−0.06–0.09)	0.14+ (−0.01–0.23)	0.16+ (−0.01–0.22)	0.09 (−0.06–0.28)	0.08 (−0.07–0.28)
Food to soothe– state–based	0.36*** (0.18–0.47)	0.28*** (0.11–0.41)	0.53*** (0.41–0.69)	0.64*** (0.50–0.74)	0.17* (0.00–0.29)	0.06 (−0.07–0.14)	−0.11 (0.14–0.04)	−0.09 (−0.16–0.05)	0.05 (−0.07–0.13)	0.01 (−0.20–0.21)	0.11 (−0.03–0.36)

^+^*p* < 0.10, *p < 0.05, **p < 0.01, ***p < 0.001. FR = infant food responsiveness, T1 = infant age 2 months, T2 = infant age 6 months, T3 = infant age 14 months. Adjusted for time invariant covariates at T1: maternal age, race/ethnicity, socioeconomic status, pre-pregnancy BMI, and infant weight-for-age z-score at birth. Adjusted for time variant covariates at their respective time points: exact infant age at visit and breastfeeding status. Time invariant variables that were significantly associated with the outcomes at later time points were also entered as covariates: pressuring-finish (pre-pregnancy BMI on pressure-finish at T2); pressuring-cereal (maternal age on pressure-cereal at T2 and maternal education on pressure-cereal at T3); restrictive-amount (maternal race/ethnicity on restriction-amount at T2, maternal age on food responsiveness at T3, and income-to-needs ratio on restriction-amount at T3); restrictive-diet quality (maternal race/ethnicity on restrictive-diet quality at T2, infant weight-for-age z score at birth on restrictive-diet quality at T3, and maternal age on infant food responsiveness at T3); food to soothe-situational (maternal education on food to soothe at T2 and maternal age on food responsiveness at T3); food to soothe-state-based (maternal age on food to soothe at T2 and food responsiveness at T3, and maternal education on food to soothe at T3).

In the separate cross-lagged models examining associations between maternal controlling feeding and infant food responsiveness, we observed several concurrent associations. At 2 months, greater pressuring to finish and state-based food to soothe (i.e., when mothers are stressed, tired, nothing else works) were both significantly associated with higher infant food responsiveness. Restrictive amount and situational food to soothe (e.g., use food to soothe baby in the grocery store, while in the car) were also marginally positively associated with higher food responsiveness (*p* < 0.10). At 6 months, greater pressuring with cereal and restrictive amount were both significantly associated with higher infant food responsiveness. At 14 months, pressuring to finish was marginally positively associated with food responsiveness and restrictive diet quality was marginally negatively associated with food responsiveness (*p* < 0.10).

In the two models examining pressuring feeding styles, results showed parent-driven cross-lagged associations with infant food responsiveness. Greater pressuring to finish was a significant predictor of increases in infant food responsiveness from 2 months to 6 months (β = 0.23, *p* = 0.004, 95% CI = 0.07-0.38) and was marginally associated with higher infant food responsiveness from 6 to 14 months (β = 0.16, *p* = 0.06, 95% CI = −0.01–0.33). Greater pressuring with cereal was a significant predictor of increases in infant food responsiveness from 6 months to 14 months (β = 0.24, *p* = 0.01, 95% CI = 0.05–0.30) and was marginally associated with higher infant food responsiveness from 2 to 6 months (β = 0.14, *p* = 0.08, 95% CI = −0.01–0.23). Additionally, we observed parent-driven cross-lagged associations between situational food to soothe and food responsiveness. Greater use of situational food to soothe at 2 months was marginally associated with higher food responsiveness at 6 months (β = 0.14, *p* = 0.07, 95% CI = −0.01–0.23), and greater use of situational food to soothe at 6 months was marginally associated with higher food responsiveness at 14 months (β = 0.16, *p* =0.06, 95% CI = −0.01–0.22).

In sum, we observed (1) prospective associations between pressuring feeding styles (pressuring to finish and pressuring with cereal) and infant food responsiveness that were exclusively parent-driven and (2) and some evidence that situational food to soothe infant distress (e.g., while in the grocery store, in the car) is associated with increases in food responsiveness across infancy.

## Discussion

This is the first study to prospectively examine associations between controlling parental feeding and child food responsiveness among children aged 2 years or less. Findings from this prospective, observational study suggest that a pressuring feeding style and the use of food to soothe infant distress may be one avenue by which children become more food responsive across infancy. The current study expands the field's understanding of reciprocal relations between controlling feeding and infant food responsiveness during a sensitive period for establishing children's appetitive behaviors.

As hypothesized, we observed unidirectional cross-lagged associations from pressuring feeding styles to infant food responsiveness. Greater pressuring to finish and pressuring with cereal predicted later increases in food responsiveness, however the effect of pressuring to finish was only statistically significant from 2 to 6 months whereas the effect of pressuring with cereal was only statistically significant from 6 to 14 months. The observed patterns of associations are in accord with the introduction to complementary foods that typically takes place between 4 and 6 months, thus providing an explanation for the emergence of pressuring with cereal as a more salient predictor of food responsiveness from 6 to 14 months in our sample. This is further evidenced by the observed increase in the pressuring with cereal mean score between 2 months (*M* = 1.77, *SD* = 0.84) and 6 months (*M* = 1.98, *SD* = 0.99). Our results build on previous mixed findings from longitudinal studies and converge with a prior study showing a prospective positive association between parental encouragement to eat and child tendency to overeat among children aged 1.5 to 2.5 years (29).

Our findings diverge from the bidirectional temporal relationship between higher pressure to eat and lower food responsiveness reported by Costa and colleagues, which was conducted across two time points when children were aged 4 to 7 years (28). Pressuring to eat among infants and toddlers may contribute to children's food-approach tendencies and push children to focus on external food cues rather than internal satiety signals (29). However, as children's food preferences strengthen and picky eating increases for many children during the toddler and preschool years, lower food responsiveness may prompt increases in encouragement to eat, which may end up being counterproductive, contributing to reduced food intake and lower interest in food (42, 43). Furthermore, the current study provides a more nuanced understanding of how different types of pressure may influence food responsiveness during infancy.

Also consistent with our hypotheses, we observed unidirectional cross-lagged associations between food to soothe and infant food responsiveness. Specifically, greater use of situational food to soothe was marginally associated with increases in food responsiveness from 2 to 6 months and 6 to 14 months. These findings build on previous research showing cross-sectional associations between the use of food to soothe and infant food responsiveness in infancy (37, 44) and one prospective study among preschool-age children showing a parent-driven effect (30). Our results are suggestive of a parent-driven effect of food to soothe on food responsiveness during infancy, particularly when food to soothe is used in situations (e.g., attending to another person, in the doctor's waiting room) in which parents may perceive that they do not have the ability or time to engage in alternative soothing strategies such as shushing or rocking their baby.

In line with prior cross-sectional research with children preschool-aged and older, we observed some evidence for concurrent correlations between a restrictive feeding style and food responsiveness (45, 46). At 6 months, higher food responsiveness was associated with greater restrictive feeding regarding food amount. This positive association between restricting food intake and food responsiveness was also apparent at 2 months and we observed an inverse correlation between restrictive feeding regarding diet quality at 12 months, though neither of these correlations reached statistical significance. Two prior studies have shown inverse longitudinal associations between restriction and food-approaching behaviors. Jansen and colleagues found that lower covert restriction (e.g., restriction the child is unaware of) predicted increases in food responsiveness from 2 to 3.7 years (47). Another study showed that restriction of food amount at 21 months predicted lower eating in the absence of hunger, an objective measure of a child's propensity to eat in response to external food cues, at 27 months (48). Our models, however, did not reveal any significant cross-lagged paths between restriction and food responsiveness, which could be explained by the lack of follow-up past 14 months in the current study. It is possible that restriction may only influence *changes* in children's food-approaching behaviors after the 1st year of life once exposure to a wider variety of foods, including less healthy energy-dense foods, becomes more common. Although evidence points to the possibility that restriction across infancy and toddlerhood may function as a protective factor, the role of restriction on the development of food-approaching behaviors remains unclear and additional research, particularly studies that measure different types of restriction (e.g., amount versus diet quality, covert vs. overt) across the first few years of life, is certainly needed.

The relative absence of a child-driven cross-lagged effect of food responsiveness on restrictive feeding in the current study did not support our hypothesis. Although a recent systematic review reported a pooled effect for a prospective positive association between food responsiveness and restriction (26), the findings from individual studies are mixed and primarily point to null longitudinal effects. Costa and colleagues found that parental perception of excess food intake (1-item adapted from the CEBQ food responsiveness scale) at age 4 predicted greater restriction 3 years later (28) whereas two other studies did not find evidence that food responsiveness influences restriction across 2-year (age 6 to 8 years) and four-year periods (age 2–4 to 5–7 years) (19, 30). In addition, child eating in the absence of hunger did not predict restriction in terms of amount or diet quality in a study conducted with toddlers (48). Taken together, there is little evidence to suggest that children's food responsiveness strongly influences maternal beliefs and behaviors surrounding how much and what types of food young children eat, and it is likely that it is children's weight, rather than their eating behaviors, that causes parental concern and subsequent restrictive feeding.

The effects observed in the current study were fairly small, which is consistent with previous research in this area (19, 26). Small effects were expected given the prospective cross-lagged models controlled for previous levels of controlling feeding and food responsiveness, which were both relatively stable (19), in addition to key covariates previously shown to influence these constructs (i.e., maternal age, race/ethnicity, pre-pregnancy BMI, socioeconomic status, infant birth weight, and breastfeeding status). Regardless, it is important to recognize the potential public health impact of controlling feeding on appetitive behaviors across infancy and early childhood. The relative stability of controlling feeding demonstrated in our study is consistent with research in older children (28, 30, 47, 48) and suggests that maternal feeding styles and practices are established by 1 year of age. This highlights the need for future research to examine factors that contribute to controlling feeding among infants, especially in the case of pressure given our results support a unidirectional parent-driven effect. The longitudinal stability of infant food responsiveness in the current study was lower than previously reported in studies conducted with children 2 years and older (27, 30, 47). Taken together, interventions that target maternal feeding after the 1st year may have limited success in modifying appetitive behaviors to reduce later obesity risk (47). Further, untested moderators may explain why several associations did not reach statistical significance and the small effects more generally. Future studies can extend this work by examining whether the tested associations depend on other child characteristics (e.g., temperament), maternal behaviors (e.g., feeding mode) or broader home environment factors (e.g., poverty status). Harris and colleagues, for example, found that the association between food responsiveness and the use of food to soothe depended on levels of negative affect and regulation (37), two dimensions of infant temperament that have been independently linked to food to soothe (15, 49).

Although the current study has several strengths, including a prospective design and adjustment for multiple covariates, our results must be considered in light of certain limitations. Although our sample was relatively diverse in terms of race/ethnicity and socioeconomic status, results may not generalize beyond a mid-sized Southeastern US city. Additionally, the sample size was somewhat small which may have reduced our ability to detect statistically significant effects. Given the small sample, we conducted separate path analysis models for each controlling feeding subscale. Further, satiety responsiveness (i.e., sensitivity to internal satiety cues) is another appetitive behavior that has been associated with infant weight in previous studies (2, 4). While we considered examining satiety responsiveness in the current study, this BEBQ subscale had low internal consistency reliability in our sample at the first two time points (αs = 0.39 and 0.47 at 2 months and 6 months respectively). Thus, we focused on food responsiveness only. Given the number of models tested in the current study, focusing on one appetitive behavior reduced the potential for Type 1 error. Studies with larger sample sizes that have the ability to test associations between food responsiveness (and other potentially important appetitive behaviors) and multiple feeding styles in one model are needed. Finally, measurement of both parental feeding and infant food responsiveness relied on parent reports, which are subject to social desirability and shared variance bias. However, the subscales reported were derived from widely used validated questionnaires and the food responsiveness subscale from the BEBQ has been validated against objective measures of appetitive behaviors (50). The potential for inflated associations due to the use of a single reporter might be reduced by the fact that the cross-lagged effects controlled for prior levels of both constructs, thus adjusting for bias in the cross-sectional paths (19). However, future longitudinal research that employs observational methods to assess parental controlling feeding is warranted.

Results from the current study reveal that pressuring, but not restrictive feeding styles, contribute to small increases in infant food responsiveness. There were no child-driven effects and our findings suggest that food responsiveness during infancy does not elicit parent's restrictive feeding. To build on trials that have had success in reducing controlling feeding, including pressuring feeding and food to soothe (51–53), qualitative studies are needed to better understand infant characteristics and contextual factors that contribute to feeding styles in order to provide more tailored messaging and support for parents in future interventions. Future work should also consider initiating feeding interventions during the prenatal period. Additional longitudinal studies that target infancy through early childhood are needed to understand how these associations unfold over time and whether child-driven effects of food responsiveness become apparent as children get older.

## Data availability statement

The raw data supporting the conclusions of this article will be made available by the authors, without undue reservation.

## Ethics statement

The studies involving human participants were reviewed and approved by University of North Carolina at Greensboro Institutional Review Board. Written informed consent to participate in this study was provided by the participants' legal guardian/next of kin.

## Author contributions

EL, CB, LS, and LW contributed to the design and measurement approaches used in the iGrow study. SE conducted the primary analyses with some assistance from EL. SE drafted the manuscript. SE and EL had primary responsibility for the final content. All authors read and approved the final manuscript, contributed to the conceptualization of this manuscript, and revised the paper.

## Funding

This study was supported by R01HD093662 from the Eunice Kennedy Shriver National Institute of Child Health and Human Development.

## Conflict of interest

The authors declare that the research was conducted in the absence of any commercial or financial relationships that could be construed as a potential conflict of interest.

## Publisher's note

All claims expressed in this article are solely those of the authors and do not necessarily represent those of their affiliated organizations, or those of the publisher, the editors and the reviewers. Any product that may be evaluated in this article, or claim that may be made by its manufacturer, is not guaranteed or endorsed by the publisher.

## Author disclaimer

The content is solely the responsibility of the authors and does not necessarily represent the official views of the Eunice Kennedy Shriver National Institute of Child Health and Human Development or the National Institutes of Health.

## References

[1] Llewellyn CH van Jaarsveld CH Johnson L Carnell S Wardle J. Development and factor structure of the baby eating behavior questionnaire in the gemini birth cohort. Appetite. (2011) 57:388–96. 10.1016/j.appet.2011.05.324 21672566

[2] van Jaarsveld CH Llewellyn CH Johnson L Wardle J. Prospective associations between appetitive traits and weight gain in infancy. Am J Clin Nutr. (2011) 94:1562–7. 10.3945/ajcn.111.015818 22071702

[3] van Jaarsveld CH Boniface D Llewellyn CH Wardle J. Appetite and growth: a longitudinal sibling analysis. JAMA Pediatr. (2014) 168:345–50. 10.1001/jamapediatrics.2013.4951 24535222

[4] Quah PL Chan YH Aris IM Pang WW Toh JY Tint MT. Prospective associations of appetitive traits at 3 and 12 months of age with body mass index and weight gain in the first 2 years of life. BMC Pediatr. (2015) 15:1–10. 10.1186/s12887-015-0467-8 26459321 PMC4603814

[5] Carnell S Wardle J. Appetitive traits in children. New evidence for associations with weight and a common, obesity-associated genetic variant. Appetite. (2009) 53:260–3. 10.1016/j.appet.2009.07.014 19635515

[6] Scaglioni S Arrizza C Vecchi F Tedeschi S. Determinants of children's eating behavior. Am J Clin Nutr. (2011) 94:2006S−11S. 10.3945/ajcn.110.001685 22089441

[7] Ashcroft J Semmler C Carnell S van Jaarsveld CH Wardle J. Continuity and stability of eating behaviour traits in children. Eur J Clin Nutr. (2008) 62:985–90. 10.1038/sj.ejcn.1602855 17684526

[8] Llewellyn CH van Jaarsveld CH Johnson L Carnell S Wardle J. Nature and nurture in infant appetite: analysis of the gemini twin birth cohort. Am J Clin Nutr. (2010) 91:1172–9. 10.3945/ajcn.2009.28868 20335548

[9] Carnell S Benson L Pryor K Driggin E. Appetitive traits from infancy to adolescence: using behavioral and neural measures to investigate obesity risk. Physiol Behav. (2013) 121:79–88. 10.1016/j.physbeh.2013.02.015 23458627 PMC3725261

[10] Birch LL Doub AE. Learning to Eat: Birth to age 2 Y. Am J Clin Nutr. (2014) 99:723S−8S. 10.3945/ajcn.113.069047 24452235

[11] Thompson AL. Intergenerational impact of maternal obesity and postnatal feeding practices on pediatric obesity. Nutr Rev. (2013) 71 Suppl 1:S55–61. 10.1111/nure.12054 24147925 PMC4123431

[12] Ventura AK Birch LL. Does parenting affect children's eating and weight status? IJBNPA. (2008) 5:1–12. 10.1186/1479-5868-5-15 18346282 PMC2276506

[13] Vaughn AE Tabak RG Bryant MJ Ward DS. Measuring parent food practices: a systematic review of existing measures and examination of instruments. IJBNPA. (2013) 10:1–27. 10.1186/1479-5868-10-61 23688157 PMC3681578

[14] DiSantis KI Hodges EA Johnson SL Fisher JO. The role of responsive feeding in overweight during infancy and toddlerhood: a systematic review. Int J Obes. (2011) 35:480–92. 10.1038/ijo.2011.3 21427696 PMC6598438

[15] Stifter CA Anzman-Frasca S Birch LL Voegtline K. Parent use of food to soothe infant/toddler distress and child weight status. An Exploratory Study Appetite. (2011) 57:693–9. 10.1016/j.appet.2011.08.013 21896298

[16] Stifter CA Moding KJ. Understanding and measuring parent use of food to soothe infant and toddler distress: a longitudinal study from 6 to 18 months of age. Appetite. (2015) 95:188–96. 10.1016/j.appet.2015.07.009 26164121 PMC4589459

[17] Jansen PW Derks IPM Batenburg A Jaddoe VWV Franco OH Verhulst FC. Using food to soothe in infancy is prospectively associated with childhood bmi in a population-based cohort. J Nutr. (2019) 149:788–94. 10.1093/jn/nxy277 30989177

[18] Ruzicka EB Darling KE Sato AF. Controlling child feeding practices and child weight: a systematic review and meta-analysis. Obes Rev. (2021) 22:e13135. 10.1111/obr.13135 32840023

[19] Steinsbekk S Belsky J Wichstrom L. Parental feeding and child eating: an investigation of reciprocal effects. Child Dev. (2016) 87:1538–49. 10.1111/cdev.12546 27154834

[20] Afonso L Lopes C Severo M Santos S Real H Durao C. Bidirectional association between parental child-feeding practices and body mass index at 4 and 7 y of age. Am J Clin Nutr. (2016) 103:861–7. 10.3945/ajcn.115.120824 26843159

[21] Eichler J Schmidt R Poulain T Hiemisch A Kiess W Hilbert A. Stability, continuity, and bi-directional associations of parental feeding practices and standardized child body mass index in children from 2 to 12 years of age. Nutrients. (2019) 11:1751. 10.3390/nu11081751 31366059 PMC6723946

[22] Hughes SO Power TG O'Connor TM Fisher JO Micheli NE Papaioannou MA. Maternal feeding style and child weight status among hispanic families with low-income levels: a longitudinal study of the direction of effects. IJBNPA. (2021) 18:1–13. 10.1186/s12966-021-01094-y 33588844 PMC7885249

[23] Jansen PW Tharner A van der Ende J Wake M Raat H Hofman A. Feeding practices and child weight: is the association bidirectional in preschool children? Am J Clin Nutr. (2014) 100:1329–36. 10.3945/ajcn.114.088922 25332330

[24] Liszewska N Scholz U Radtke T Horodyska K Luszczynska A. Bi-directional associations between parental feeding practices and children's body mass in parent-child dyads. Appetite. (2018) 129:192–7. 10.1016/j.appet.2018.07.011 30017947

[25] Tschann JM Martinez SM Penilla C Gregorich SE Pasch LA de Groat CL. Parental feeding practices and child weight status in mexican american families: a longitudinal analysis. IJBNPA. (2015) 3:1–10. 10.1186/s12966-015-0224-2 25986057 PMC4453102

[26] Wang J Zhu B Wu R Chang YS Cao Y Zhu D. Bidirectional associations between parental non-responsive feeding practices and child eating behaviors: a systematic review and meta-analysis of longitudinal prospective studies. Nutrients. (2022) 14:1896. 10.3390/nu14091896 35565862 PMC9103127

[27] Papaioannou MA Micheli N Power TG O'Connor TM Fisher JO Hughes SO. Maternal feeding styles and child appetitive traits: direction of effects in hispanic families with low incomes. Public Health Front. (2022) 10:1550. 10.3389/fpubh.2022.871923 35719648 PMC9201210

[28] Costa A Severo M Oliveira A. Food parenting practices and eating behaviors in childhood: a cross-lagged approach within the generation Xxi cohort. Am J Clin Nutr. (2021) 114:101–8. 10.1093/ajcn/nqab024 33742190

[29] Rodgers RF Paxton SJ Massey R Campbell KJ Wertheim EH Skouteris H. Maternal feeding practices predict weight gain and obesogenic eating behaviors in young children: a prospective study. IJBNPA. (2013) 10:1–10. 10.1186/1479-5868-10-24 23414332 PMC3582584

[30] Berge JM Miller J Veblen-Mortenson S Kunin-Batson A Sherwood NE French SA. Bidirectional analysis of feeding practices and eating behaviors in parent/child dyads from low-income and minority households. J Pediatr. (2020) 221:93–8. 10.1016/j.jpeds.2020.02.001 32247517 PMC7252585

[31] Ekelund U Ong KK Linne Y Neovius M Brage S Dunger DB. Association of weight gain in infancy and early childhood with metabolic risk in young adults. J Clin Endocrinol Metab. (2007) 92:98–103. 10.1210/jc.2006-1071 17032722

[32] Taveras EM Rifas-Shiman SL Bettylou S. Crossing growth percentiles in infancy and risk of obesity in childhood. JAMA Pediatr. (2011) 165:993–8. 10.1001/archpediatrics.2011.167 22065180

[33] Thompson AL Mendez MA Borja JB Adair LS Zimmer CR Bentley ME. Development and validation of the infant feeding style questionnaire. Appetite. (2009) 53:210–21. 10.1016/j.appet.2009.06.010 19576254 PMC3130353

[34] Lumeng JC Kaciroti N Retzloff L Rosenblum K Miller AL. Longitudinal associations between maternal feeding and overweight in low-income toddlers. Appetite. (2017) 113:23–9. 10.1016/j.appet.2017.02.016 28212827 PMC5382094

[35] Rogers SL Blissett J. Infant temperament, maternal feeding behaviors and the timing of solid food introduction. Matern Child Nutr. (2019) 15:e12771. 10.1111/mcn.12771 30560584 PMC7198933

[36] Ventura AK Pollack Golen R A. Pilot study comparing opaque, weighted bottles with conventional, clear bottles for infant feeding. Appetite. (2015) 85:178–84. 10.1016/j.appet.2014.11.028 25445988 PMC4309547

[37] Harris HA Moore AM Ruggiero CF Bailey-Davis L Savage JS. Infant food responsiveness in the context of temperament and mothers' use of food to soothe. Front Nutr. (2022) 8:1265. 10.3389/fnut.2021.781861 35087856 PMC8786708

[38] Shriver LH Eagleton SG Lawless M. C. Buehler C Wideman L LEerkes EM. Infant appetite and weight gain in early infancy: moderating effects of controlling feeding styles. Appetite. (2022) 176:106139. 10.1016/j.appet.2022.106139 35718312 PMC12040430

[39] Poverty Thresholds: United States Census Bureau. Available from: https://www.census.gov/data/tables/time-series/demo/income-poverty/historical-poverty-thresholds.html (accessed March 29, 2021).

[40] Fein SB Labiner-Wolfe J Shealy KR Li R Chen J Grummer-Strawn LM. Infant feeding practices study II: study methods. Pediatrics. (2008) 122 Suppl 2:S28–35. 10.1542/peds.2008-1315c 18829828

[41] Acock AC. Working with missing values. J Marriage Fam. (2005) 67:1012–28. 10.1111/j.1741-3737.2005.00191.x

[42] Jansen PW de Barse LM Jaddoe VWV Verhulst FC Franco OH Tiemeier H. Bi-directional associations between child fussy eating and parents' pressure to eat: who influences whom? Physiol Behav. (2017) 176:101–6. 10.1016/j.physbeh.2017.02.015 28215424 PMC5436628

[43] Galloway AT Fiorito LM Francis LA Birch LL. 'Finish your soup': counterproductive effects of pressuring children to eat on intake and affect. Appetite. (2006) 46:318–23. 10.1016/j.appet.2006.01.019 16626838 PMC2604806

[44] Mallan KM Sullivan SE de Jersey SJ Daniels LA. The relationship between maternal feeding beliefs and practices and perceptions of infant eating behaviors at 4 months. Appetite. (2016) 105:1–7. 10.1016/j.appet.2016.04.032 27133549

[45] Jansen PW Roza SJ Jaddoe VW Mackenbach JD Raat H Hofman A. Children's eating behavior, feeding practices of parents and weight problems in early childhood: results from the population-based generation R study. IJBNPA. (2012) 130:1–11. 10.1186/1479-5868-9-130 23110748 PMC3543222

[46] Webber L Cooke L Hill C Wardle J. Associations between children's appetitive traits and maternal feeding practices. J Am Diet Assoc. (2010) 110:1718–22. 10.1016/j.jada.2010.08.007 21034886

[47] Jansen E Williams KE Mallan KM Nicholson JM Daniels LA. Bidirectional associations between mothers' feeding practices and child eating behaviors. IJBNPA. (2018) 15:1–11. 10.1186/s12966-018-0644-x 29325557 PMC5765660

[48] Bauer KW Haines J Miller AL Rosenblum K Appugliese DP Lumeng JC. Maternal restrictive feeding and eating in the absence of hunger among toddlers: a cohort study. Int J Behav Nutr Phys Act. (2017) 14:1–10. 10.1186/s12966-017-0630-8 29258621 PMC5735902

[49] Stifter CA Moding KJ. Infant temperament and parent use of food to soothe predict change in weight-for-length across infancy: early risk factors for childhood obesity. Int J Obes. (2018) 42:1631–8. 10.1038/s41366-018-0006-4 29463917 PMC6066452

[50] Carnell S Wardle J. Measuring behavioral susceptibility to obesity: validation of the child eating behaviour questionnaire. Appetite. (2007) 48:104–13. 10.1016/j.appet.2006.07.075 16962207

[51] Savage JS Hohman EE Marini ME Shelly A Paul IM Birch LL. Insight responsive parenting intervention and infant feeding practices: randomized clinical trial. Int J Behav Nutr Phys Act. (2018) 15:64. 10.1186/s12966-018-0700-6 29986721 PMC6038199

[52] Ruggiero CF Hohman EE Birch LL Paul IM Savage JS. The intervention nurses start infants growing on healthy trajectories (insight) responsive parenting intervention for firstborns impacts feeding of secondborns. Am J Clin Nutr. (2020) 111:21–7. 10.1093/ajcn/nqz277 31782493 PMC6944525

[53] Daniels LA Mallan KM Nicholson JM Battistutta D Magarey A. Outcomes of an early feeding practices intervention to prevent childhood obesity. Pediatrics. (2013) 132:e109–18. 10.1542/peds.2012-2882 23753098

